# Pancreatic Plasmacytoma: A Case of Recurrent Disease

**DOI:** 10.7759/cureus.26502

**Published:** 2022-07-01

**Authors:** Bola Nashed, Arshan Khan, Mohamed Issa, Laura Kohler, Mohammed Barawi

**Affiliations:** 1 Internal Medicine, Ascension St. John Hospital, Detroit, USA; 2 Hematology and Medical Oncology, Ascension St. John Hospital, Detroit, USA; 3 Gastroenterology, Ascension St. John Hospital, Detroit, USA

**Keywords:** pancreatic lesions, pancreatic cancer, obstructive jaundice, ercp, endoscopic utrasound, endoscopic ultrasound-guided fine-needle aspiration

## Abstract

Pancreatic plasmacytoma is a rare entity of extramedullary plasmacytomas (EMP). It is important to consider pancreatic plasmacytoma in patients diagnosed with multiple myeloma (MM) presenting with obstructive jaundice. We present a case of pancreatic plasmacytoma in a patient with previously diagnosed multiple myeloma and extramedullary plasmacytoma in remission. Endoscopic ultrasound (EUS) and endoscopic retrograde cholangiopancreatography (ERCP) were of great diagnostic and therapeutic value for acute management.

## Introduction

Extramedullary plasmacytomas (EMP) are a rare entity of hematological plasma cell malignancies (PCM) that affect soft tissues [1]. EMPs can be primary or secondary, depending on bone marrow involvement and serological markers [2]. They occur in about 5% of patients with multiple myeloma (MM) and are usually present as a progression of their underlying disease [3]. EMPs mostly affect the head, neck, and upper respiratory tract; however, in 10% of the patients, they affect the gastrointestinal tract-primarily the liver, spleen, or stomach [4,5]. Pancreatic plasma cell neoplasms are a rare entity in EMPs [5]. They usually occur in the head of the pancreas, resulting in obstructive jaundice and abdominal pain [2,6]. In this clinical vignette, we describe a patient with a history of multiple myeloma who relapsed with pancreatic extramedullary plasmacytoma.

## Case presentation

Our patient is a 62-year-old male with a history of multiple myeloma with EMPs. He was initially diagnosed in 2011 after presenting with abdominal pain and weight loss. Imaging at that time showed massively enlarged mesenteric lymph nodes. Biopsy confirmed lambda-restricted plasmacytoma.

After work-up, including a bone marrow biopsy, he was diagnosed with multiple myeloma. The large lymph nodes were not amenable to radiation. He was subsequently treated with five cycles of lenalidomide-bortezomib-dexamethasone (RVd) with a good response. He then continued lenalidomide as maintenance therapy. In March 2021, his monoclonal protein began to rise, consistent with disease progression. PET/CT at that time showed a 6 cm × 9.3 cm mass in the region of the head of the pancreas.

**Figure 1:**
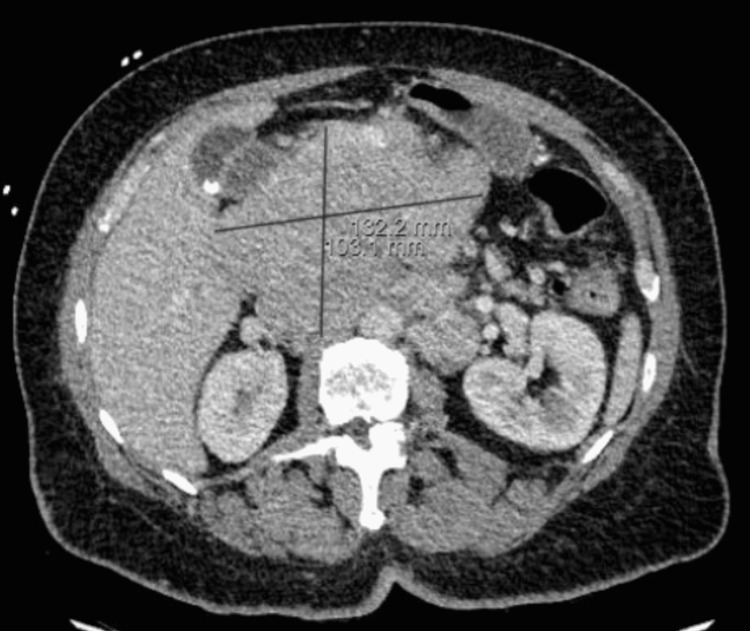
Pancreatic head mass Axial CT scan abdomen and pelvis with IV contrast showing irregular shaped, minimally enhancing solid large pancreatic head lesion measuring 13.2 cm × 10.3 cm

After that, he started on daratumumab, lenalidomide, and dexamethasone. Three months later, the patient presented to the hospital with back pain and obstructive jaundice. The CT scan of the abdomen showed the pancreatic head mass larger than before, causing biliary obstruction and showing enlarged retroperitoneal lymphadenopathy (Figure 1).

Endoscopic ultrasound (EUS) showed a hypoechoic heterogeneous mass in the head of the pancreas. An EUS-guided fine-needle aspiration (FNA) of the mass was performed and showed fibrosis and chronic inflammation. Due to external compression of the mass on the common bile duct (CBD), a sphincterotomy with the placement of a 7-French × 7 cm double-pigtail stent was performed.

The patient improved initially but had a recurrence of jaundice two weeks later. Repeat endoscopy with stent repositioning and repeat pancreatic head FNA was obtained, showing lambda-restricted monoclonal plasma cells consistent with plasmacytoma. Symptoms improved again, and he was discharged home. He subsequently received radiation of 2400 cGy to the mass and then restarted systemic therapy.

## Discussion

Pancreatic involvement as an EMP is very rare and few cases are reported in the literature. About 80% of pancreatic EMPs are located in the head of the pancreas [2]. Obstructive jaundice is the most common presenting symptom, followed by abdominal pain [7].

Pancreatic EMPs can be visualized in several imaging modalities. CT typically shows a well-defined, lobulated, homogeneous soft-tissue mass that is low-attenuating [8]. In patients with a solitary plasmacytoma, a PET/CT scan should be obtained to exclude the involvement of other organs if a whole-body MRI is unavailable [9]. They may also be repeated for progression or recurrence, or to assess response to therapy [10].

EUS is increasingly used for the biopsy and staging of pancreatic masses. It has detection rates of 99-100% for all pancreatic carcinomas, including those smaller than 3 cm, which are often missed by transcutaneous US and CT [2]. EUS is often used along with endoscopic retrograde cholangiopancreatography (ERCP) for CBD stenting in patients presenting with obstructive jaundice. The demonstration of smooth stenosis of the biliary tree is more suggestive of an EMP than adenocarcinoma, where irregular stenosis is classical [11].

A definitive diagnosis is through biopsy. In a systematic review of 63 patients with pancreatic plasmacytoma, 14 patients were diagnosed with EUS FNA, 9 patients were diagnosed through CTguided FNA, 11 patients were diagnosed through surgical biopsy, and 3 patients were diagnosed with a postmortem autopsy [7]. Cytologic diagnosis can be made in 85-95% of patients with pancreatic masses via EUS FNA, making it the most effective means for a definitive diagnosis [2]. A bone marrow (BM) sample is also essential to clarify whether the EMP occurs in the context of a PCM, as this will have an effect on therapy [2]. The concurrent presence of clonal plasma cells in BM and EMP makes the diagnosis of PCM, and the EMP is regarded as secondary.

EMPs are extremely radiosensitive tumors. Therefore, the modality of choice is radiation over 4-6 weeks [12]. An alternative strategy for tumors larger than 5 cm is chemotherapy followed by radiotherapy [13]. Mignot et al. reported that concurrent use of lenalidomide-dexamethasone with intensity-modulated radiation therapy in the treatment of EMPs improved multiple myeloma-free survival and progression-free survival [14]. Small lesions may be treated with surgical resection alone [15].

Our patient had a pancreatic plasmacytoma in the setting of progressive multiple myeloma. Evaluations included CT and EUS with biopsy. He was treated with radiation at a dose of 24 Gy. He was also restarted on systemic therapy with RVd, as he refused alternative second- and third-line options.

## Conclusions

In conclusion, pancreatic extramedullary plasmacytomas are rare. They often present similarly to other pancreatic tumors and require tissue diagnosis. Radiation therapy is the first-line treatment. This can be combined with systemic therapy if multiple myeloma is also present. This approach may improve multiple myeloma-free survival and progression-free survival as compared to radiation therapy alone.
